# Supplementary material for: Dietary supplementation of arachidonic acid promotes humoral immunity
Source: EMBO Mol Med. 2025 Sep 12;17(11):2966–94. doi: 10.1038/s44321-025-00310-7 (PMC12603062; doi:10.1038/s44321-025-00310-7)
Supplement: Supplementary file 1 — Appendix [file 44321_2025_310_MOESM1_ESM.pdf]

Appendix Data

Table of contents

Contents	Page
Appendix Figure S1	2
Appendix Figure S2	3
Appendix Figure S3	4
Appendix Table S1	5
Appendix Table S2	6
Appendix Table S3	7
Appendix Table S4	8
Appendix Table S5	9-10
Appendix Table S6	11
Appendix Table S7	12
Appendix Table S8	13
Appendix Table S9	14

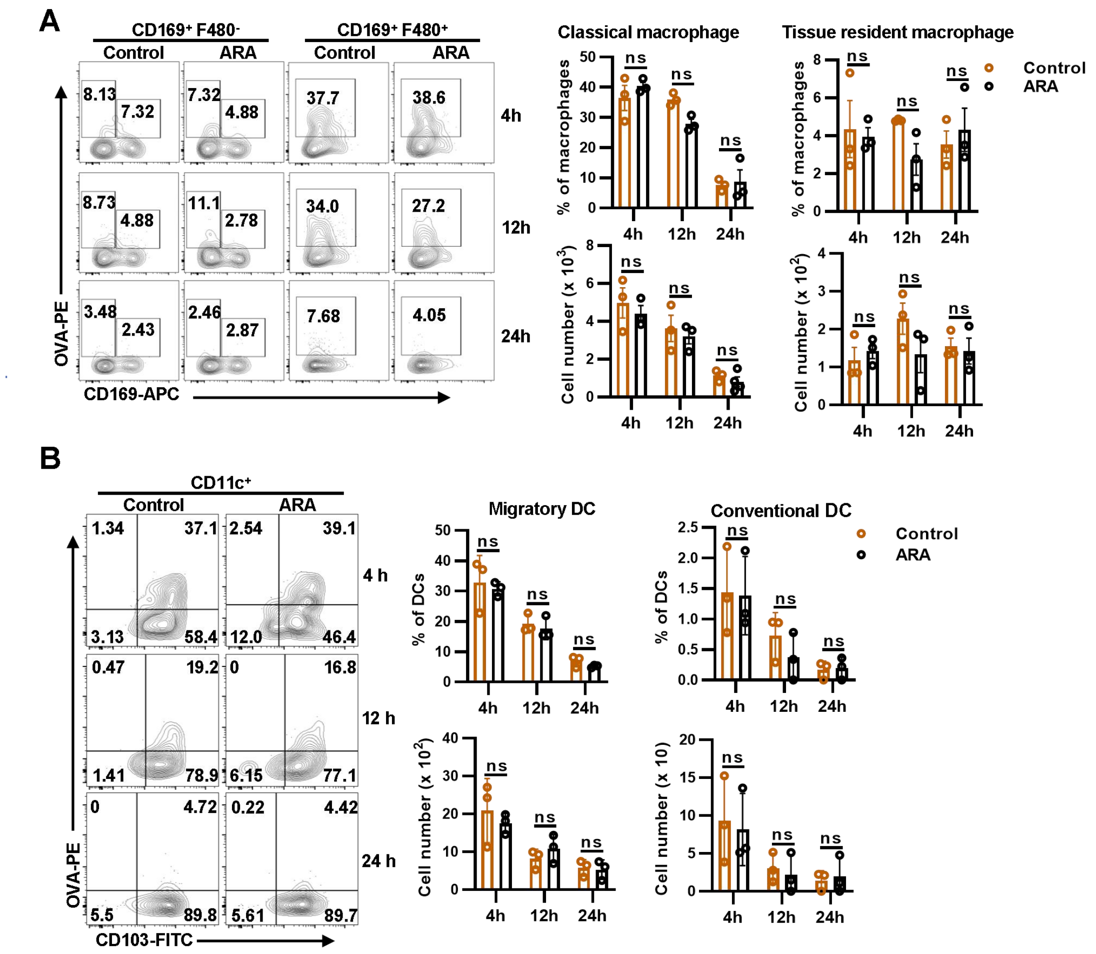

Appendix Figure S1. Oral administration of ARA did not affect antigen presentation, CD4 and CD8 T cells.

(A and B) Mice were immunized with PE-OVA. The draining lymph nodes were collected 4, 12, and 24 hours after immunization. PE-positive macrophages (A) and dendritic cells (B) were analyzed by flow cytometry (n=3). Left: Representative flow cytometry plots. Right: Statistic data of the percentages and cell numbers of the corresponding cells. Data are representative of two independent experiments. Data are shown as mean ± SEM and each point represents an individual mouse. Significance was calculated by unpaired two-tailed t test; ns, no statistical significance.

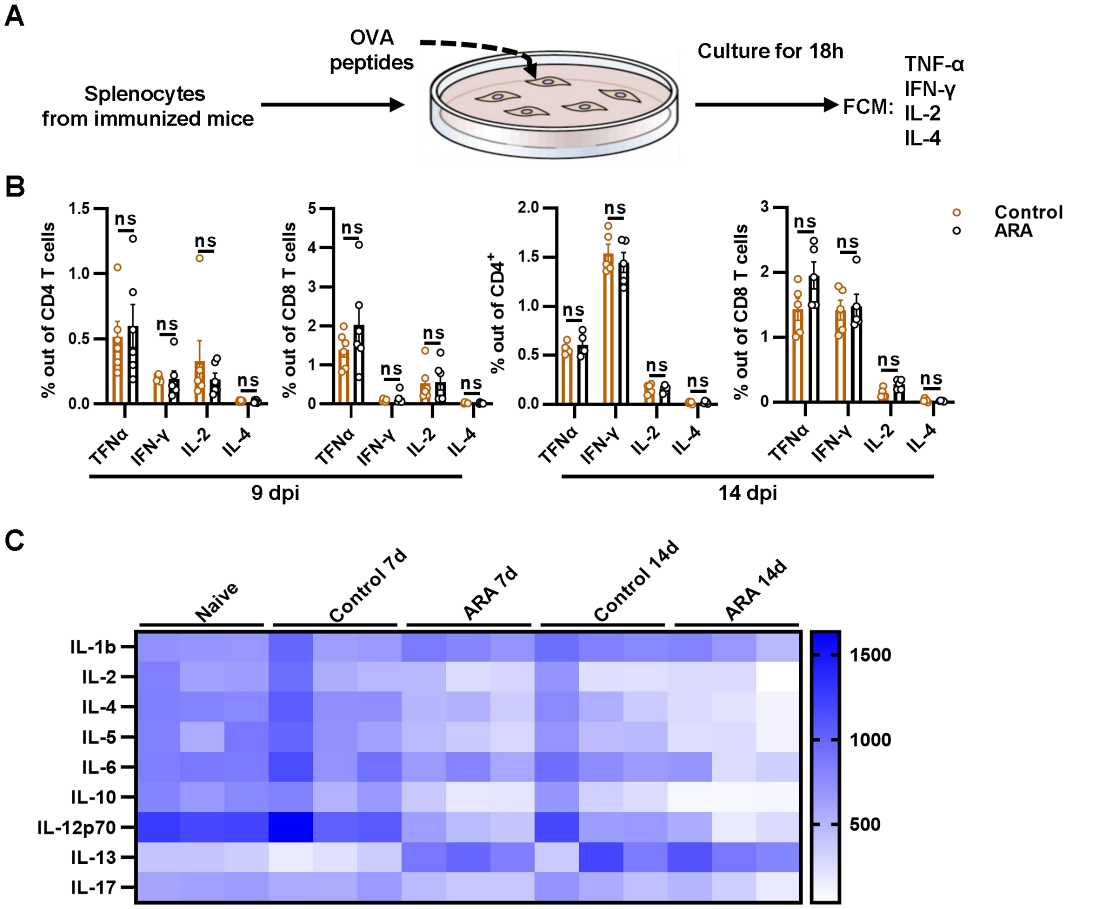

Appendix Figure S2. Supplementation of ARA did not regulate the secretion of cytokines. (A) Schematic diagram of the study design. Splenocytes of mice immunized with OVA were subjected to restimulation with OVA peptides for 18 hours to detect the T cells secreting cytokines. (B) Flow cytometry analysis of T cells secreting various cytokines from mice that i.m. immunized with OVA on day 9 (left) and day 14 (right) (n = 6). (C) Analysis of cytokines from the serum of mice immunized with OVA on day 7 and day 14 through Luminex assay (n = 3). Data are representative of two independent experiments. Data are shown as mean ± SEM and each point represents an individual mouse. Significance was calculated by unpaired two-tailed t test; ns, no statistical significance.

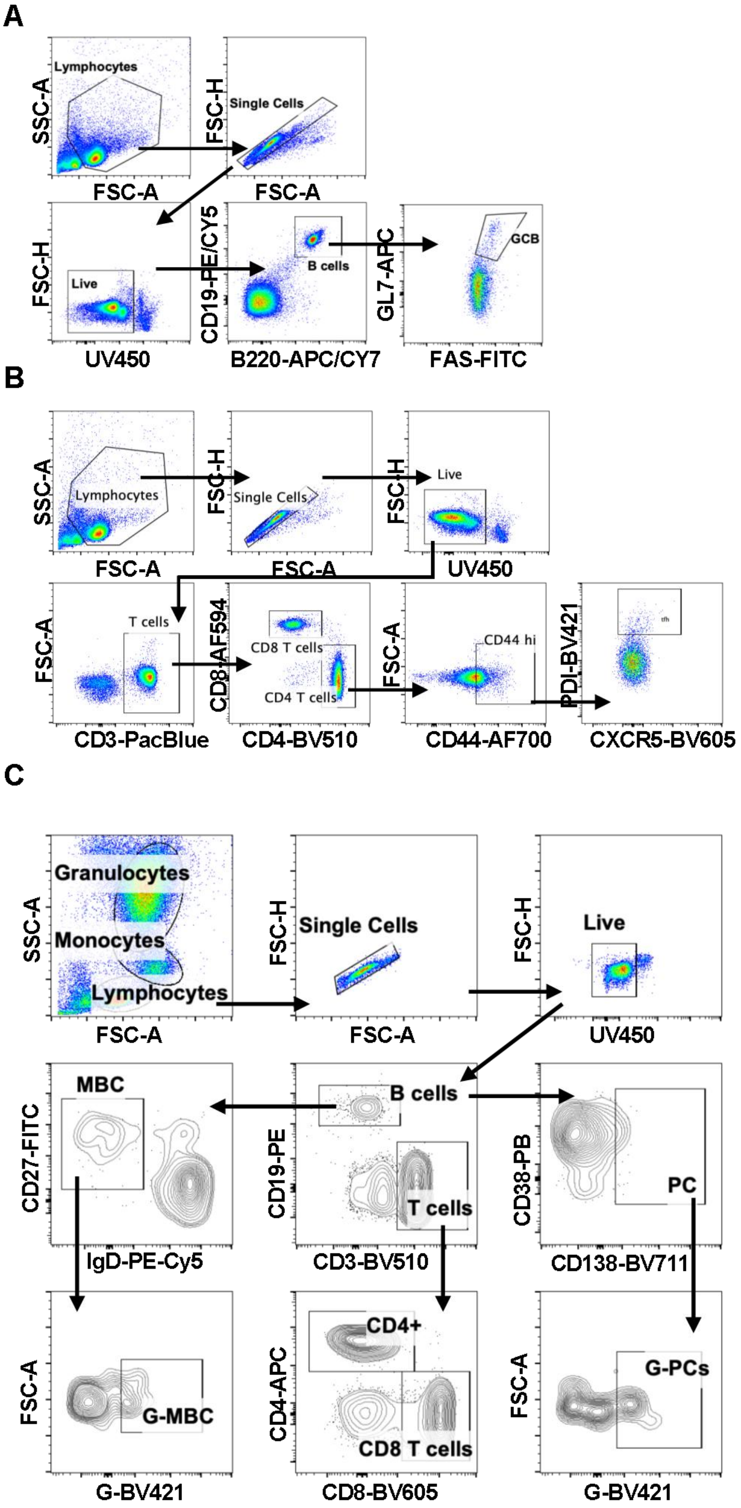

Appendix Figure S3. FCM gating strategy.

(A) Gating strategy to identify GCB cells in the inguinal LNs of mice. (B) Gating strategy to identify Tfh in the inguinal LNs of mice. (C) Gating strategy to identify antigen specific memory B cells and plasma cells in volunteer PBMCs.

Appendix Table S1. Blood routine index of mice supplemented with ARA.

**Physiological indicators**	**Control (n=6, mean ± SD)**	**ARA (n=6, mean ± SD)**	**Reference**
WBC (10^9/L)	6.1±0.36	6.08±0.27	0.8-6.8
Lymph (10^9/L)	4.93±0.3	4.67±0.55	0.7-5.7
Mon (10^9/L)	0.22±0.07	0.2±0.06	0.0-0.3
Gran (10^9/L)	1.3±0.14	1.38±0.13	0.1-1.8
RBC (10^12/L)	9.14±0.09	9.18±0.1	6.36-9.42
HGB (g/L)	135.5±4.15	132.17±5.52	110-143
HCT (%)	43.12±0.74	43.02±0.8	34.6-44.6
MCV (fL)	49.48±0.7	49.82±0.76	48.2-58.3
MCH (pg)	16.58±0.7	16.98±0.71	15.8-19
MCHC (g/L)	323.67±6.26	330.67±6.82	302-353
RDW (%)	15.12±0.45	15.15±0.56	13-17
PLT (10^9/L)	529.5±68.98	498.67±37.67	450-1590
MPV (fL)	5.35±0.24	5.48±0.25	3.8-6.0

Abbreviations: SD, standard deviation; WBC, white blood cell; Lymph, lymphocyte; Mon, monocyte; Gran, granulocyte; RBC, red blood cell; HGB, haemoglobin; HCT, hematocrit; MCV, mean corpusular volume; MCH, mean corpusular hemoglobin; MCHC, mean corpusular hemoglobin concerntratio; RDW, red blood cell volume distribution width; PLT, blood platelet count; MPV, mean platelet volume.

Appendix Table S2. List of standards for targeted mass spectrometry.

Eicosanoids	Abbreviations
(±)14,15-Epoxyeicosatrienoic acid	(±)14(15)-EET
(±)17-Hydroxyeicosatetraenoic acid	(±)17-HETE
(±)18-Hydroxyeicosatetraenoic acid	(±)18-HETE
12(R)-Hydroxyeicosatetraenoic acid	12(R)-HETE
8(R)-Hydroxyeicosatetraenoic acid	8(R)-HETE
(±)9-Hydroxyeicosatetraenoic acid	(±)9-HETE
11(R)-Hydroxyeicosatetraenoic acid	11(R)-HETE
(±)5,6-Epoxyeicosatrienoic acid	(±)5(6)-EET
15(S)-Hydroxyeicosatetraenoic acid	15(S)-HETE
16(R)-Hydroxyeicosatetraenoic acid	16(R)-HETE
(±)8(9)-Epoxyeicosatrienoic acid	(±)8(9)-EET
(±)11(12)-Epoxyeicosatrienoic acid	(±)11(12)-EET
Prostaglandin D_2	PGD_2
Prostaglandin E_2	PGE_2
Leukotriene E_4	LTE_4
Leukotriene D_4	LTD_4
(±)5-Hydroxyeicosatetraenoic acid	(±)5-HETE
6-keto-Prostaglandin $F_{1\alpha}$	6-keto-$PGF_{1\alpha}$
15-Deoxy-Δ-12,14-prostaglandin J_2	15d-PGJ_2
Leukotriene B_4	LTB_4
Thromboxane B_2	TxB_2
15-keto-Prostaglandin E_2	15-keto-PGE_2
Prostaglandin $F_{2\alpha}$	$PGF_{2\alpha}$
Leukotriene C_4	LTC_4
Anandamide	AEA

Appendix Table S3. The abundance of eicosanoids in sera from mice.

Eicosanoid	Control (n=6, mean ± SD)	ARA (n=6, mean ± SD)
TXB_2	3826.18 ± 2011.26	2022.98 ± 467.73
$PGF_{2\alpha}$	3999.31 ± 2045.48	3375.86 ± 1264.58
5-HETE	2739.10 ± 1815.92	4107.18 ± 1199.01
9-HETE	2585.07 ± 1514.47	2941.06 ± 1058.33
11-HETE	3527.57 ± 1074.41	3803.81 ± 1731.42
12-HETE	50191.22 ± 32239.92	34931.11 ± 24371.32
15-HETE	3053.55 ± 975.67	2848.36 ± 1106.55
17-HETE	3651376.51 ± 891649.64	3149664.01 ± 831236.35
18-HETE	3651376.51 ± 891649.64	3149664.01 ± 831236.35
15d-PGJ_2	NA	NA
8-HETE	NA	NA
16-HETE	NA	NA
17-HETE	NA	NA
5.6-EET	NA	NA
8.9-EET	NA	NA
11.12-EET	NA	NA
14.15-EET	NA	NA
AEA	NA	NA
LTE_4	NA	NA
LTB_4	NA	NA
LTD_4	NA	NA
6-keto-$PGF_{1\alpha}$	NA	NA
15-keto-PGE_2	NA	NA
PGD_2	NA	NA
PGE_2	NA	NA

Abbreviations: SD, standard deviation; NA, more than 3 samples that not detect the eicosanoid in the group.

Appendix Table S4. Demographic characteristics and lifestyles at baseline among three groups.

	**Placebo** n=14	**ARA** n=15	**Pre-ARA** n=15	***P***
Age, years, mean±SD	24.43±3.13	24.87±2.97	23.80±1.42	0.508
Sex				0.212
Male	8 (57.1)	5 (33.3)	4 (26.7)	
Female	6 (42.9)	10 (66.7)	11 (73.3)	
Income, n (%)				0.273
<2,000 Chinese yuan/month	7 (50.0)	5 (33.3)	11 (73.3)	
2000~4999 Chinese yuan/month	4 (28.6)	6 (40.0)	3 (20.0)	
>5,000 Chinese yuan/month	3 (21.4)	4 (26.7)	1 (6.67)	
BMI, kg/m^2, mean±SD	21.33±1.84	21.59±2.20	21.79±1.57	0.513
Physical activity, MET-min/week, median (Q_1, Q_3)	1576 (604, 2260)	1426 (1013, 2470)	1426 (1192, 3501)	0.707
Sleep time, hours/day, mean±SD	7.96±0.73	7.98±0.81	7.82±0.75	0.616
Alcohol consumption, n (%)				0.537
Never	3 (21.4)	6 (40.0)	8 (53.3)	
Seldom	9 (64.3)	7 (46.7)	6 (40.0)	
>once/month	2 (14.3)	2 (13.3)	1 (6.67)	
Health situation, n (%)				0.484
OK	3 (21.4)	7 (46.7)	4 (26.7)	
Good	10 (71.4)	7 (46.7)	8 (53.3)	
Very healthy	1 (7.14)	1 (6.67)	3 (20.0)	

Abbreviations: Pre-ARA, taking 512.4 mg of arachidonic acid daily on Day -3–13; ARA, taking 512.4 mg of arachidonic acid daily on Day 0–13; SD, standard deviation; Q_1, lower quartile; Q_3, upper quartile.

Appendix Table S5. Dietary intake during intervention among three groups.

Energy/ macronutrient	Placebo group n=14	ARA group n=15	Pre-ARA group n=15	*P*
Energy intake, kcal/day, median (Q_1, Q_3)				
day-6-day-4	1742.79 (1446.64, 2340.16)	2094.19 (1614.69, 2776.30)	1482.88 (1194.38, 2027.93)	0.127
day5-day7	1829.04 (1580.40, 2274.50)	2245.86 (1789.72, 2918.51)	2074.53 (1268.15, 2234.97)	0.255
day11-day13	1786.75 (1550.92, 2426.56)	2181.52 (1699.22, 2912.67)	1751.37 (1327.28, 2043.78)	0.241
Carbohydrate, g/day, median (Q_1, Q_3)				
day-6-day-4	172.00 (151.85, 268.62)	242.84 (190.11, 419.56)	140.68 (112.69, 217.50)	0.064
day5-day7	206.97 (148.66, 309.99)	267.78 (192.79, 415.85)	233.58 (112.02, 292.78)	0.279
day11-day13	197.63 (129.49, 251.54)	228.57 (182.87, 403.18)	146.61 (129.68, 268.32)	0.284
Protein, g/day, median (Q_1, Q_3)				
day-6-day-4	61.62 (50.86, 75.41)	57.33 (44.01, 82.54)	50.51 (41.09, 62.96)	0.417
day5-day7	58.91 (49.37, 85.57)	77.05 (60.81, 96.42)	65.14 (51.84, 78.67)	0.184
day11-day13	69.64 (52.74, 79.50)	69.27 (56.73, 104.44)	67.26 (44.23, 85.75)	0.717
Fat, g/day, median (Q_1, Q_3)				
day-6-day-4	77.92 (70.25, 83.34)	73.87 (59.52, 83.73)	72.09 (61.88, 77.97)	0.370
day5-day7	74.49 (69.45, 86.18)	78.26 (67.63, 100.91)	73.23 (67.50, 80.81)	0.600
day11-day13	76.13 (70.20, 90.04)	85.93 (66.16, 93.76)	76.66 (65.10, 91.54)	0.857
PUFA, g/day, median (Q_1, Q_3)				
day-6-day-4	21.36 (20.08, 23.11)	20.09 (18.18, 21.95)	19.64 (19.09, 21.87)	0.293
day5-day7	23.15 (20.58, 25.39)	21.72 (18.60, 23.83)	22.74 (20.29, 25.01)	0.560
day11-day13	20.65 (19.35, 24.71)	19.41 (18.80, 22.67)	21.50 (18.83, 23.01)	0.632
20:4n-6, mg/day, median (Q_1, Q_3)				
day-6-day-4	253.63 (126.14, 293.35)	123.29 (73.96, 177.78)	155.36 (86.13, 202.14)	0.059
day5-day7	206.19 (169.18, 357.80)	212.39 (108.86, 243.12)	198.80 (136.68, 269.65)	0.708
day11-day13	262.08 (173.13, 324.92)	175.95 (131.57, 223.40)	195.15 (141.18, 311.79)	0.280
18:2n-6, g/day, median (Q_1, Q_3)				
day-6-day-4	25.41 (16.32, 54.65)	38.62 (20.91, 65.48)	38.10 (18.85, 50.60)	0.485
day5-day7	48.21 (32.93, 71.68)	50.05 (34.40, 67.08)	20.43 (16.74, 44.76)	0.237
day11-day13	36.61 (28.94, 41.18)	22.92 (19.24, 56.96)	18.17 (12.02, 43.56)	0.319
18:3n-3, g/day, median (Q_1, Q_3)				
day-6-day-4	4.08 (2.65, 10.32)	6.25 (3.62, 11.17)	6.96 (3.15, 9.28)	0.651
day5-day7	7.91 (5.65, 10.25)	7.36 (4.87, 12.92)	3.47 (2.75, 7.32)	0.147
day11-day13	6.27 (4.44, 7.02)	4.28 (3.52, 9.27)	3.04 (1.90, 7.34)	0.175
20:5n-3, mg/day, median (Q_1, Q_3)				
day-6-day-4	8.95 (2.63, 12.10)	22.15 (5.70, 96.17)	14.72 (3.98, 24.25)	0.333
day5-day7	18.36 (11.16, 46.68)	23.80 (16.77, 57.73)	38.30 (13.27, 78.84)	0.726
day11-day13	55.94 (13.58, 90.52)	9.40 (7.37, 78.59)	45.40 (13.28, 104.41)	0.512
22:6n-3, mg/day, median (Q_1, Q_3)				
day-6-day-4	129.00 (63.88, 208.58)	98.60 (27.55, 139.36)	89.33 (54.48, 188.90)	0.519
day5-day7	143.54 (84.85, 297.92)	164.47 (99.36, 282.37)	182.38 (62.45, 290.79)	0.974
day11-day13	154.36 (136.44, 317.97)	89.59 (51.07, 222.60)	178.73 (103.08, 230.47)	0.331

Abbreviations: Pre-ARA, taking 512.4 mg of arachidonic acid daily on Day -3–13; ARA, taking 512.4 mg of

arachidonic acid daily on Day 0–13. PUFA, polyunsaturated fatty acid; ARA, arachidonic acid; 18:2n-6, linoleic acid; 18:3n-3, α-linolenic acid; 20:5n-3, eicosapentaenoic acid; 22:6n-3, docosahexaenoic acid.

Appendix Table S6. The relative abundance of eicosanoids derived from ARA in volunteer plasma.

**Eicosanoid**	**Placebo (n=14, mean ± SD)**	**ARA (n=15, mean ± SD)**	**Pre-ARA (n=15, mean ± SD)**
TXB_2	39054.60 ±31876.40	53560.15 ±25344.39	61526.82 ±53361.74
$PGF_{2\alpha}$	19206.94 ±7070.18	21768.87 ±5127.05	18345.57 ±4203.31
PGE_2	5275.17 ±2476.74	6159.73 ±4240.60	4793.67 ±1678.63
18-HETE	15343.97 ± 5063.81	14878.56 ± 4054.77	18915.55 ± 6742.50
17-HETE	7711.81 ±2137.56	7275.63 ±2076.17	9178.85 ±2600.27
16-HETE	18740.51 ±4072.68	19111.16 ±4960.29	20773.47 ±3492.72
15-HETE	47091.38 ± 13150.85	60556.28 ± 22393.09	59866.19 ± 24653.31
11-HETE	76481.55 ±28731.43	84540.42 ±37653.24	75917.99 ±34898.72
12-HETE	1507885.31 ± 1423393.83	2083177.38 ±1048151.96	2498220.67±1859892.15
8-HETE	31320.00 ±9827.77	35724.09 ±14426.87	38171.45 ±11101.14
9-HETE	11374.71±4146.56	11902.20 ±6330.09	11474.20 ±4196.82
14.15-EET	3920.52 ±934.59	4128.67 ±617.44	4716.50 ±1451.33
LTB_4	15178.49 ±10081.52	31878.66 ±26409.33	16519.11 ±11310.97
AEA	55242.93 ±20204.39	51398.29 ±16064.85	54416.93 ±21667.84
PGD_2	7532.37 ±2039.28	10821.54 ±6618.60	NA
6-keto-$GF_{1\alpha}$	NA	NA	NA
15d-PGJ_2	NA	NA	NA
15-keto-GE_2	NA	NA	NA
11.12-EET	NA	NA	NA
5-HETE	NA	NA	NA
LTE_4	NA	NA	NA
5.6-EET	NA	NA	NA
LTD_4	NA	NA	NA
8.9-EET	NA	NA	NA

Abbreviations: SD, standard deviation; NA, more than 12 samples that not detect eicosanoid in the group.

Appendix Table S7. Changes in safety outcomes among three groups.

Physiological indicators	Placebo group n=14	ARA n=15	Pre-ARA n=15	*P*
TG, mmol/L, mean±SD	-0.03±0.34	-0.05±0.42	-0.02±0.18	0.961
TC, mmol/L, mean±SD	-0.12±0.42	-0.08±0.74	-0.46±0.61	0.185
HDL-C, mmol/L, median (Q_1, Q_3)	0.00 (-0.10, 0.05)	-0.08 (-0.15, 0.04)	-0.03 (-0.11, 0.05)	0.715
LDL-C, mmol/L, mean±SD	0.07±0.27	0.16±0.56	-0.14±0.40	0.155
CRP, mg/L, median (Q_1, Q_3)	-0.15 (-0.50, 0.07)	0.10 (-2.10, 0.40)	0.00 (-0.16, 0.10)	0.780
Platelet count, $\times 10^9$/L, mean±SD	-13.43±23.4	2.53±37.1	-14.33±38.9	0.326
PT, s, mean±SD	0.48±0.56	0.61±0.63	0.55±0.52	0.818
APTT, s, mean±SD	0.16±1.09	0.51±1.04	-0.15±1.08	0.252
TT, s, median (Q_1, Q_3)	0.75 (0.02, 1.73)	0.20 (-0.35, 1.00)	-0.30 (-0.65, 0.45)	0.041
PT%, %, mean±SD	-5.34±10.7	-10.99±8.46	-9.51±7.65	0.229
PT-INR, mean±SD	0.04±0.05	0.06±0.05	0.04±0.05	0.413
FIB, g/L, mean±SD	-0.22±0.25	-0.22±0.49	0.07±0.36	0.075

Abbreviations: Pre-ARA, taking 512.4 mg of arachidonic acid daily on Day -3–13; ARA, taking 512.4 mg of arachidonic acid daily on Day 0–13. SD, standard deviation; Q_1, lower quartile; Q_3, upper quartile; TG, triglyceride; TC, total cholesterol; HDL-C, high-density lipoprotein cholesterol; LDL-C, low-density lipoprotein cholesterol; CRP, C-reactive protein; PT, prothrombin time; APTT, activated partial thromboplastin time; TT, thromboplastin time; PT%, prothrombin time percentage activity; PT-INR, international normalized ratio of prothrombin time; FIB, fibrinogen.

Appendix Table S8. Dietary recommendation for ARA-restricted diet.

<table>
<tr><th>Food groups</th><th>Dietary Recommendation</th></tr>
<tr><td>Eggs</td><td><1/day（about 60g）</td></tr>
<tr><td>Purtenance</td><td><20g/week</td></tr>
<tr><td>Lamb</td><td rowspan="5"><75g/week</td></tr>
<tr><td>Poultry meat</td></tr>
<tr><td>Pork</td></tr>
<tr><td>Beef</td></tr>
<tr><td>Other meats</td></tr>
<tr><td>Marine fish</td><td><75g/week</td></tr>
<tr><td>Milk</td><td><300mL/day</td></tr>
</table>

Appendix Table S9. Fatty acids composition of placebo and arachidonic acid capsule.

Fatty acids	Placebo (%)	Arachidonic acid capsule (%)
C14:0	0.09	0.15
C16:0	6.12	6.08
C16:1	0.03	0.15
C18:0	3.50	4.57
C18:1	28.06	19.65
C18:2	59.40	38.46
C18:3	/	0.87
C20:0	0.27	0.50
C20:3	/	2.00
C20:4	/	18.66
C22:0	0.76	1.81
C24:0	0.26	4.05
