# Supplementary figures and images for: Dietary supplementation of arachidonic acid promotes humoral immunity
Source: EMBO Mol Med. 2025 Sep 12;17(11):2966–94. doi: 10.1038/s44321-025-00310-7 (PMC12603062; doi:10.1038/s44321-025-00310-7)

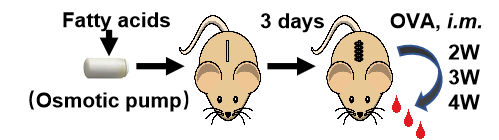

Supplement: Supplementary file 3 — Source data Fig. 1 [file 44321_2025_310_MOESM3_ESM.zip › Figure 1/1A/Schematic diagram of the study design.tif]

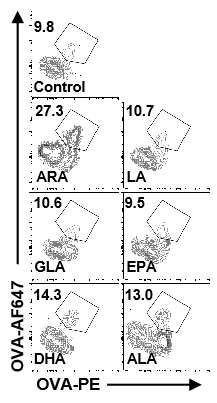

Supplement: Supplementary file 3 — Source data Fig. 1 [file 44321_2025_310_MOESM3_ESM.zip › Figure 1/1C/Representative flow cytometry plots of OVA-specific plasma cells in LN.tif]

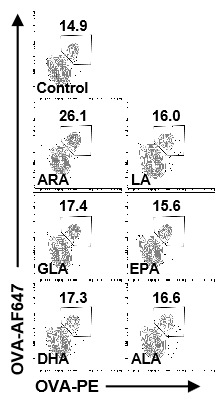

Supplement: Supplementary file 3 — Source data Fig. 1 [file 44321_2025_310_MOESM3_ESM.zip › Figure 1/1D/Representative flow cytometry plots of OVA-specific plasma cells in spleen.tif]

**Control**

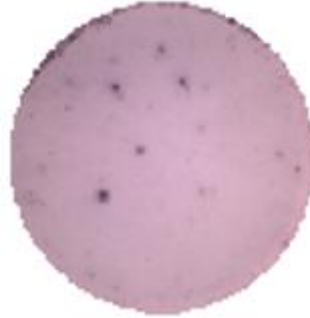

**ARA**

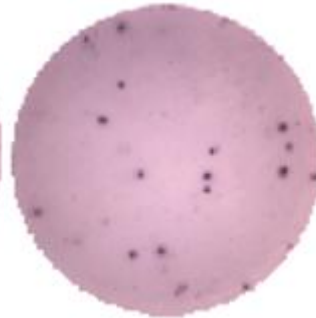

**LA**

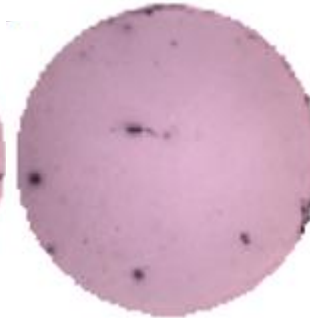

**GLA**

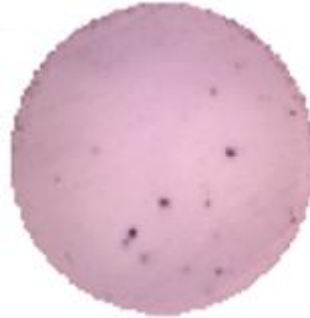

**EPA**

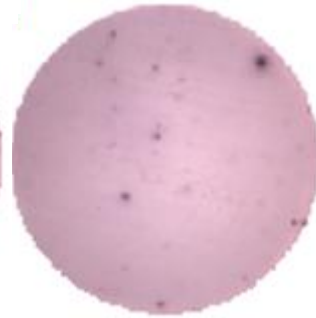

**DHA**

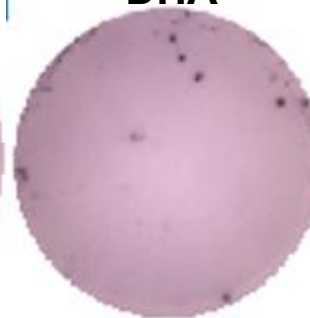

**ALA**

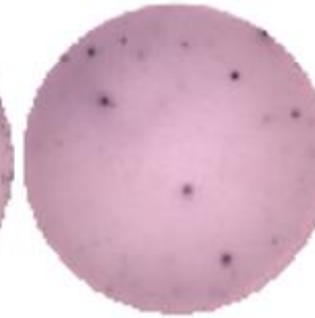

Supplement: Supplementary file 3 — Source data Fig. 1 [file 44321_2025_310_MOESM3_ESM.zip › Figure 1/1E/Fig 1E.pdf]

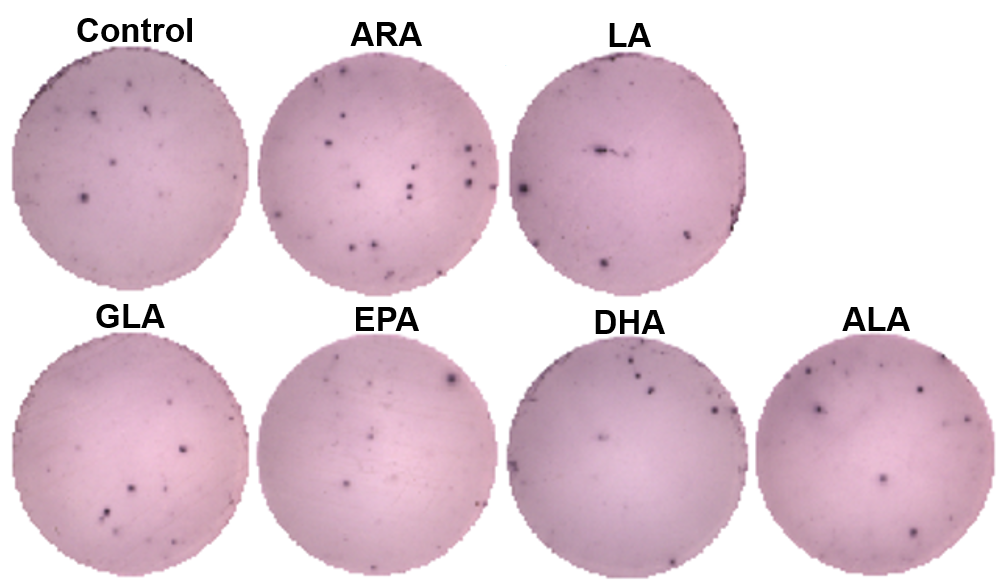

Supplement: Supplementary file 3 — Source data Fig. 1 [file 44321_2025_310_MOESM3_ESM.zip › Figure 1/1E/Representative ELISPOT plots.tif]

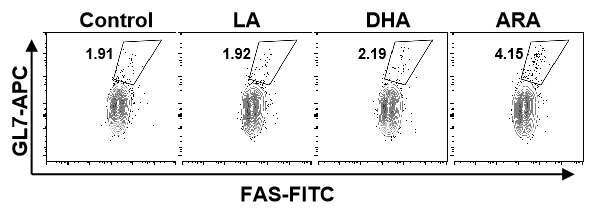

Supplement: Supplementary file 3 — Source data Fig. 1 [file 44321_2025_310_MOESM3_ESM.zip › Figure 1/1G/Representative flow cytometry plots of GC B cells.tif]

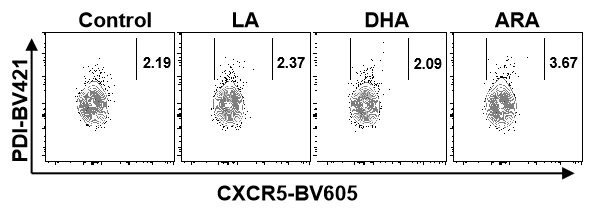

Supplement: Supplementary file 3 — Source data Fig. 1 [file 44321_2025_310_MOESM3_ESM.zip › Figure 1/1H/Representative flow cytometry plots of Tfh cells.tif]

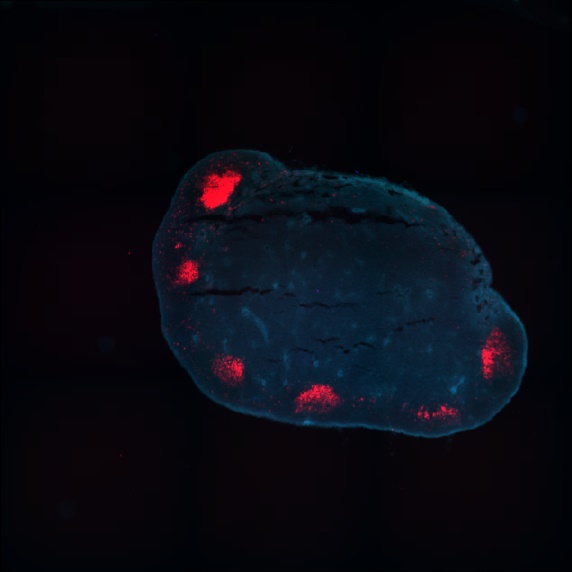

Supplement: Supplementary file 3 — Source data Fig. 1 [file 44321_2025_310_MOESM3_ESM.zip › Figure 1/1I/ARA.jpg]

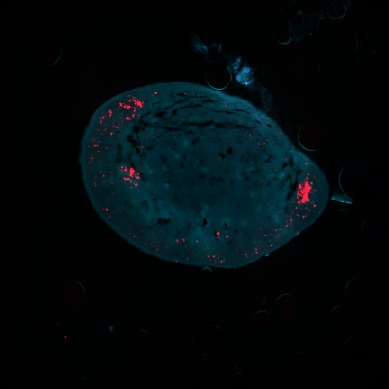

Supplement: Supplementary file 3 — Source data Fig. 1 [file 44321_2025_310_MOESM3_ESM.zip › Figure 1/1I/Control.jpg]

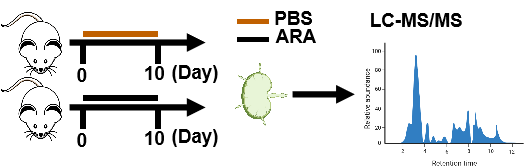

Supplement: Supplementary file 4 — Source data Fig. 2 [file 44321_2025_310_MOESM4_ESM.zip › Figure 2/2A/Schematic diagram of the study design.tif]

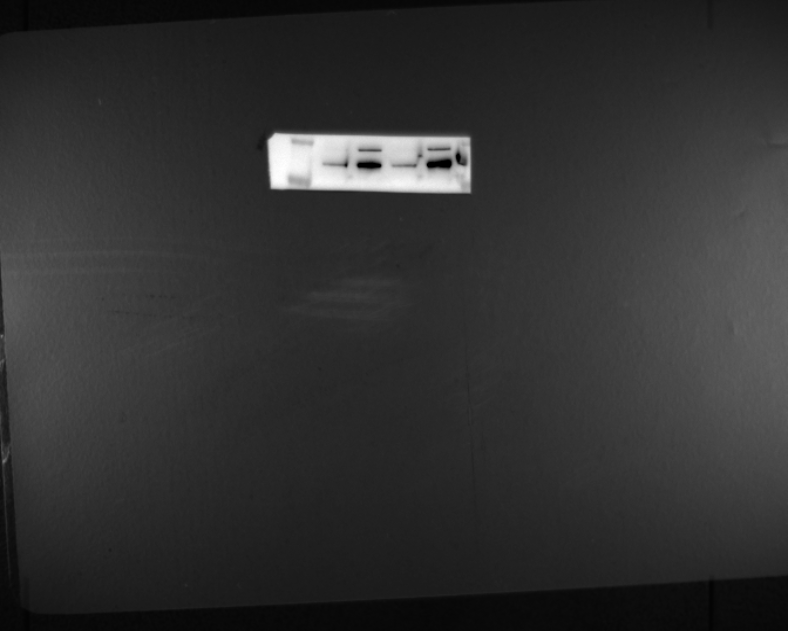

Supplement: Supplementary file 4 — Source data Fig. 2 [file 44321_2025_310_MOESM4_ESM.zip › Figure 2/2G/western-CD86.tif]

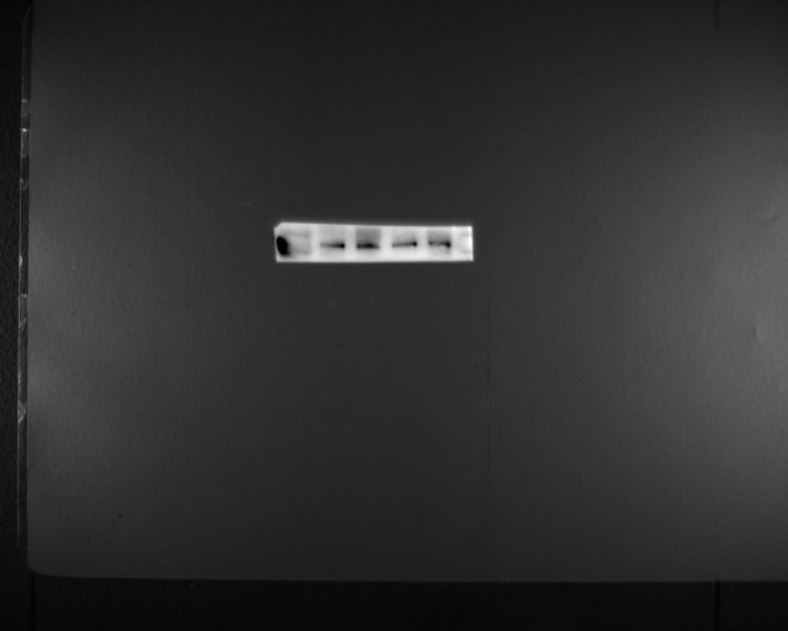

Supplement: Supplementary file 4 — Source data Fig. 2 [file 44321_2025_310_MOESM4_ESM.zip › Figure 2/2G/western-gapdh.tif]

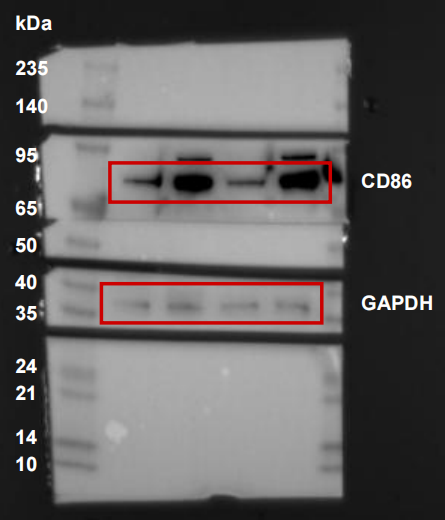

Supplement: Supplementary file 4 — Source data Fig. 2 [file 44321_2025_310_MOESM4_ESM.zip › Figure 2/2G/western-Merge1.tif]

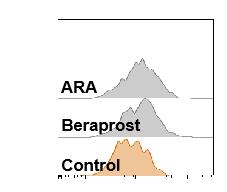

Supplement: Supplementary file 4 — Source data Fig. 2 [file 44321_2025_310_MOESM4_ESM.zip › Figure 2/2H/Representative flow cytometry plots.tif]

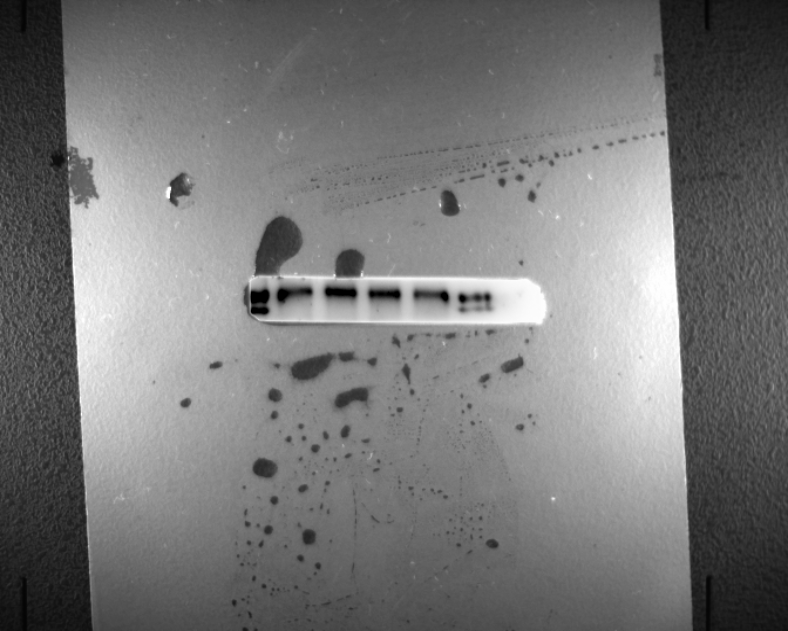

Supplement: Supplementary file 4 — Source data Fig. 2 [file 44321_2025_310_MOESM4_ESM.zip › Figure 2/2I/western-input-AID.tif]

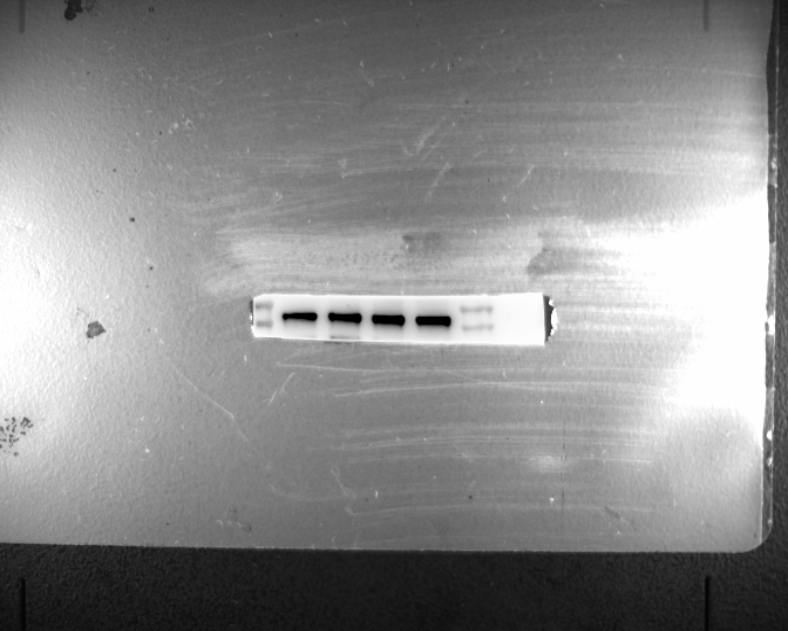

Supplement: Supplementary file 4 — Source data Fig. 2 [file 44321_2025_310_MOESM4_ESM.zip › Figure 2/2I/western-input-GAPDH.tif]

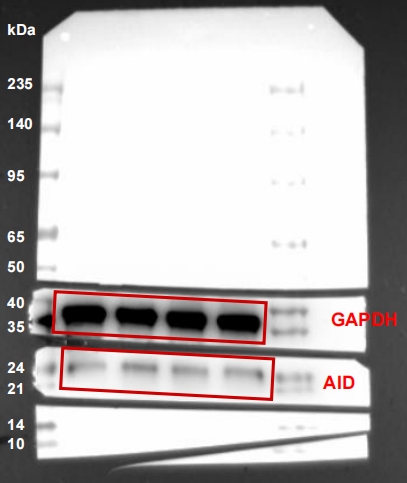

Supplement: Supplementary file 4 — Source data Fig. 2 [file 44321_2025_310_MOESM4_ESM.zip › Figure 2/2I/western-input-merge.tif]

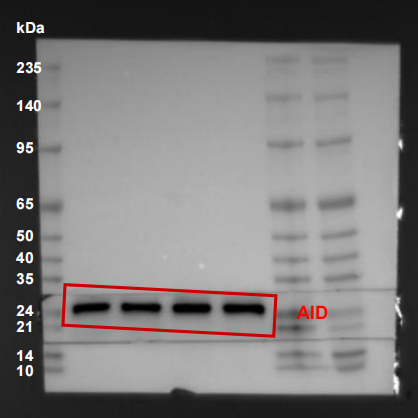

Supplement: Supplementary file 4 — Source data Fig. 2 [file 44321_2025_310_MOESM4_ESM.zip › Figure 2/2I/western-IP-AID-merge.tif]

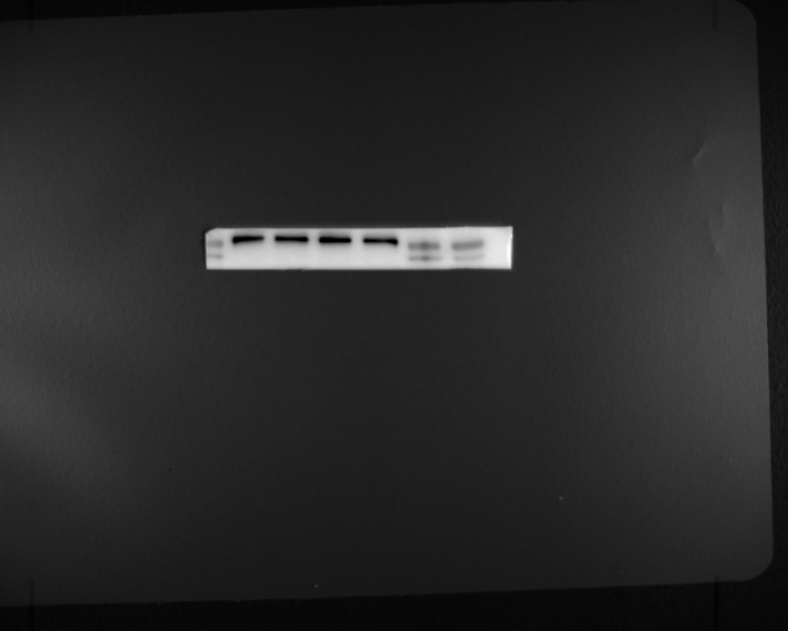

Supplement: Supplementary file 4 — Source data Fig. 2 [file 44321_2025_310_MOESM4_ESM.zip › Figure 2/2I/western-IP-AID.tif]

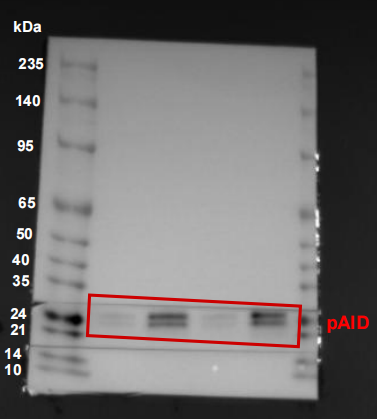

Supplement: Supplementary file 4 — Source data Fig. 2 [file 44321_2025_310_MOESM4_ESM.zip › Figure 2/2I/western-pAID-merge.tif]

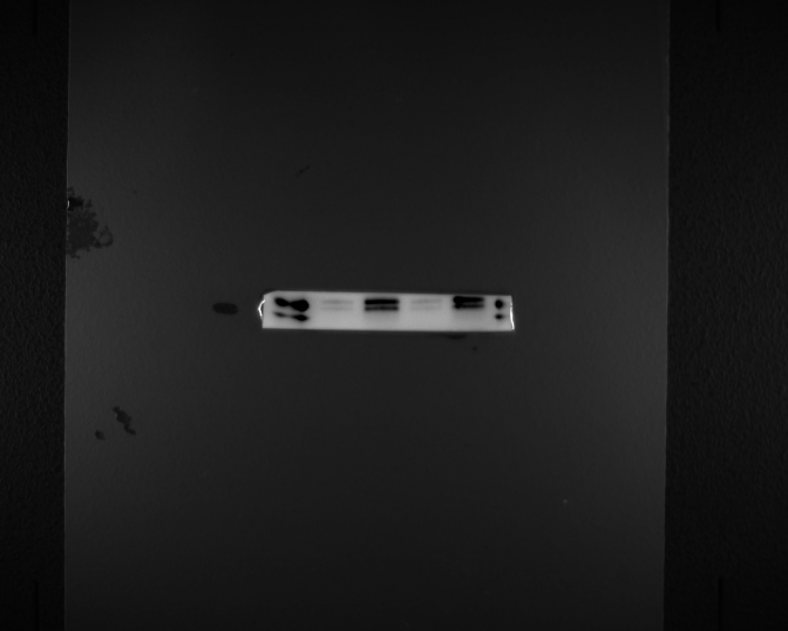

Supplement: Supplementary file 4 — Source data Fig. 2 [file 44321_2025_310_MOESM4_ESM.zip › Figure 2/2I/western-pAID.tif]

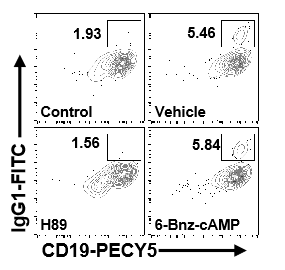

Supplement: Supplementary file 4 — Source data Fig. 2 [file 44321_2025_310_MOESM4_ESM.zip › Figure 2/2J/Representative flow cytometry plots of CD19+ IgG1+ B cells..tif]

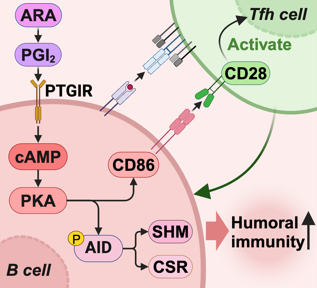

Supplement: Supplementary file 4 — Source data Fig. 2 [file 44321_2025_310_MOESM4_ESM.zip › Figure 2/2L/A mechanical scheme of supplementing ARA to promote humoral immunity.tif]

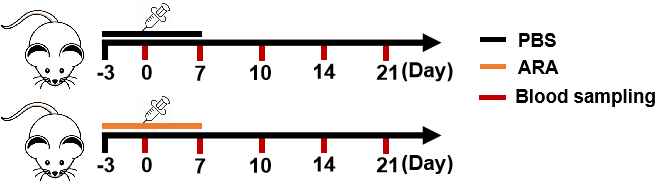

Supplement: Supplementary file 5 — Source data Fig. 3 [file 44321_2025_310_MOESM5_ESM.zip › Figure 3/3A/Schematic diagram of the study design.tif]

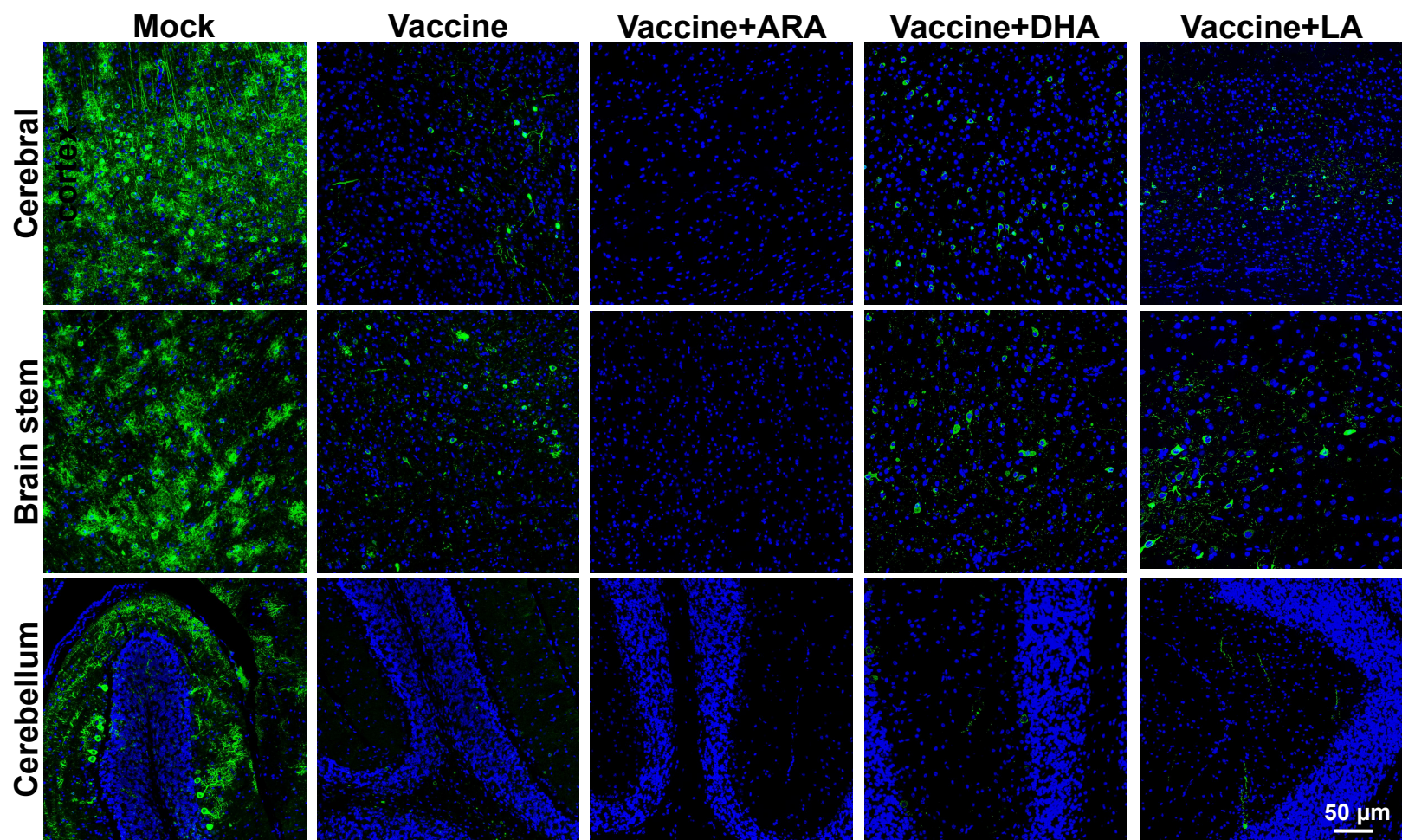

Supplement: Supplementary file 5 — Source data Fig. 3 [file 44321_2025_310_MOESM5_ESM.zip › Figure 3/3H/Immunofluorescence stain of cerebral cortex, brainstem and cerebellum.pdf]

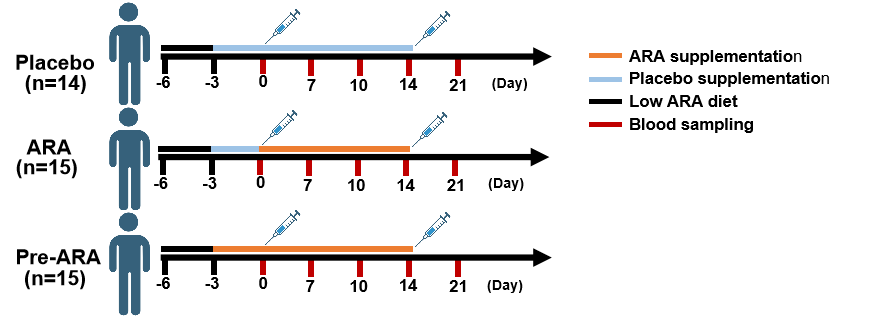

Supplement: Supplementary file 6 — Source data Fig. 4 [file 44321_2025_310_MOESM6_ESM.zip › Figure 4/4A/Schematic diagram of the study design.tif]

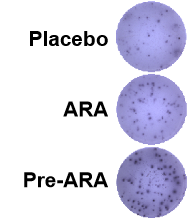

Supplement: Supplementary file 6 — Source data Fig. 4 [file 44321_2025_310_MOESM6_ESM.zip › Figure 4/4E/Representative images of ELISPOT assays.tif]

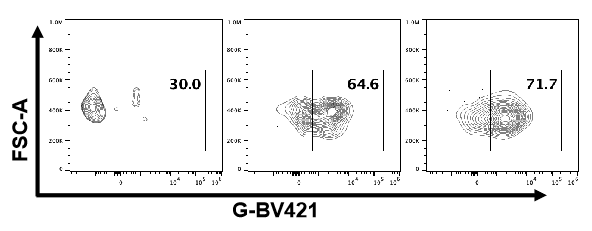

Supplement: Supplementary file 6 — Source data Fig. 4 [file 44321_2025_310_MOESM6_ESM.zip › Figure 4/4F-G/F-Flow cytometry analysis of RABV-specific specific PCs.tif]

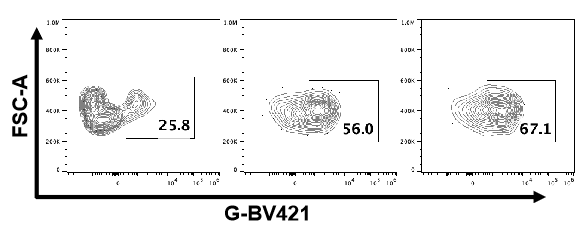

Supplement: Supplementary file 6 — Source data Fig. 4 [file 44321_2025_310_MOESM6_ESM.zip › Figure 4/4F-G/G-Flow cytometry analysis of RABV-specific MBCs.tif]
