# Supplementary material for: Dietary supplementation of arachidonic acid promotes humoral immunity
Source: EMBO Mol Med. 2025 Sep 12;17(11):2966–94. doi: 10.1038/s44321-025-00310-7 (PMC12603062; doi:10.1038/s44321-025-00310-7)
Supplement: Supplementary file 7 — Expanded View Figures [file 44321_2025_310_MOESM7_ESM.pdf]

Expanded View Figures

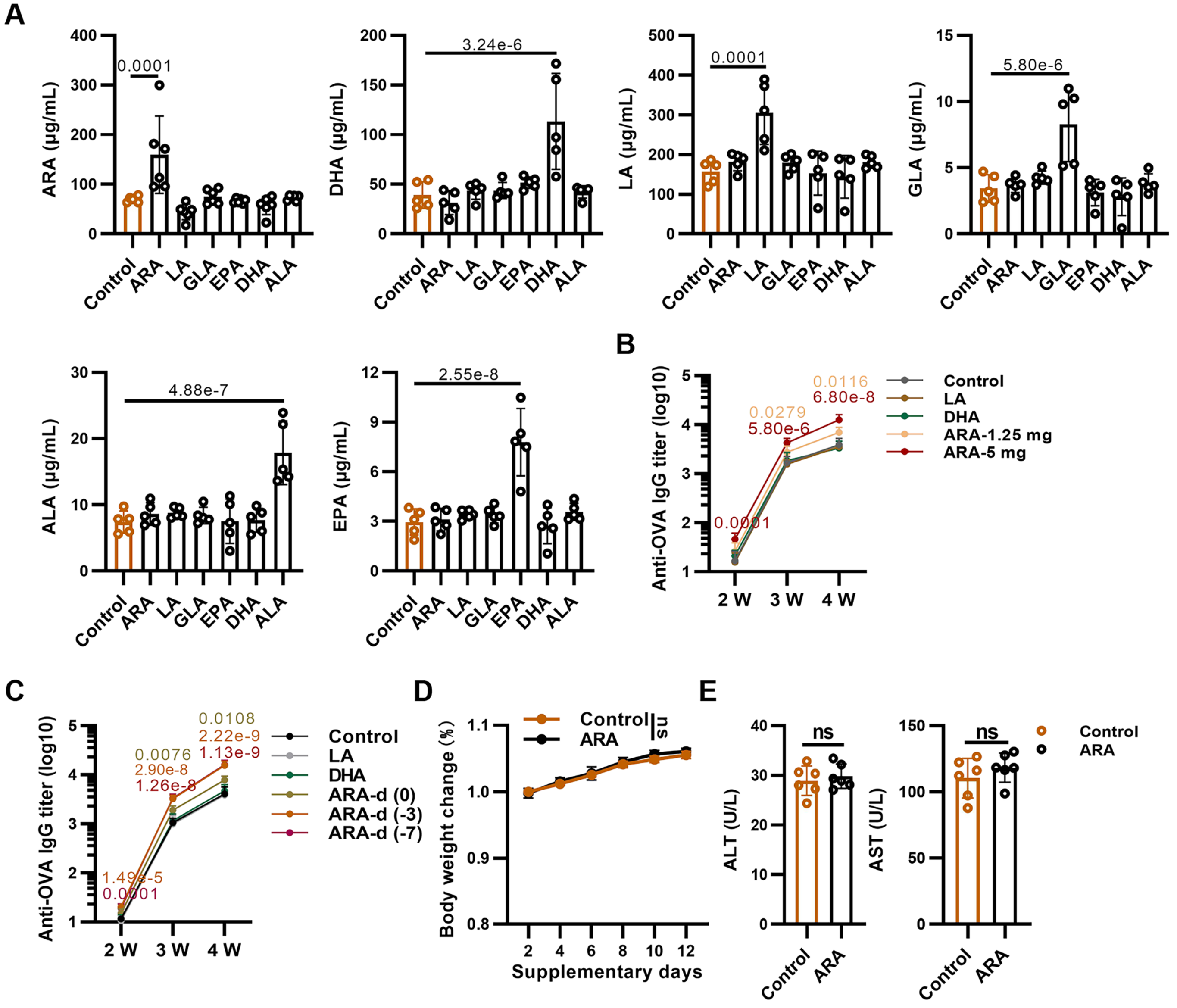

Figure EV1. Exploring optimal settings for oral supplementation of ARA.

(**A**) Fatty acid composition in murine plasma following supplementation with various PUFAs ($n = 6$ or $n = 5$). (**B**) OVA-specific IgG titers in mice orally administered serial doses of ARA at 2, 3, and 4 weeks post-immunization with OVA. DHA and LA were supplemented as unrelated controls ($n = 6$). (**C**) OVA-specific IgG titers in mice subjected to varying durations of ARA administration prior to immunization. DHA and LA were supplemented as unrelated controls ($n = 6$). (**D**) Changes in body weight of mice during ARA supplementation. (**E**) The levels of alanine aminotransferase (ALT) and aspartate aminotransferase (AST) of the mice supplemented with ARA under the optimal oral supplementation settings ($n = 6$). Data are representative of two independent experiments. Data are shown as mean ± SEM and each point represents an individual mouse. Significance was calculated by one-way ANOVA with Tukey's multiple comparisons test (**A–C**) and unpaired two-tailed *t* test (**D**, **E**); ns, no statistical significance.

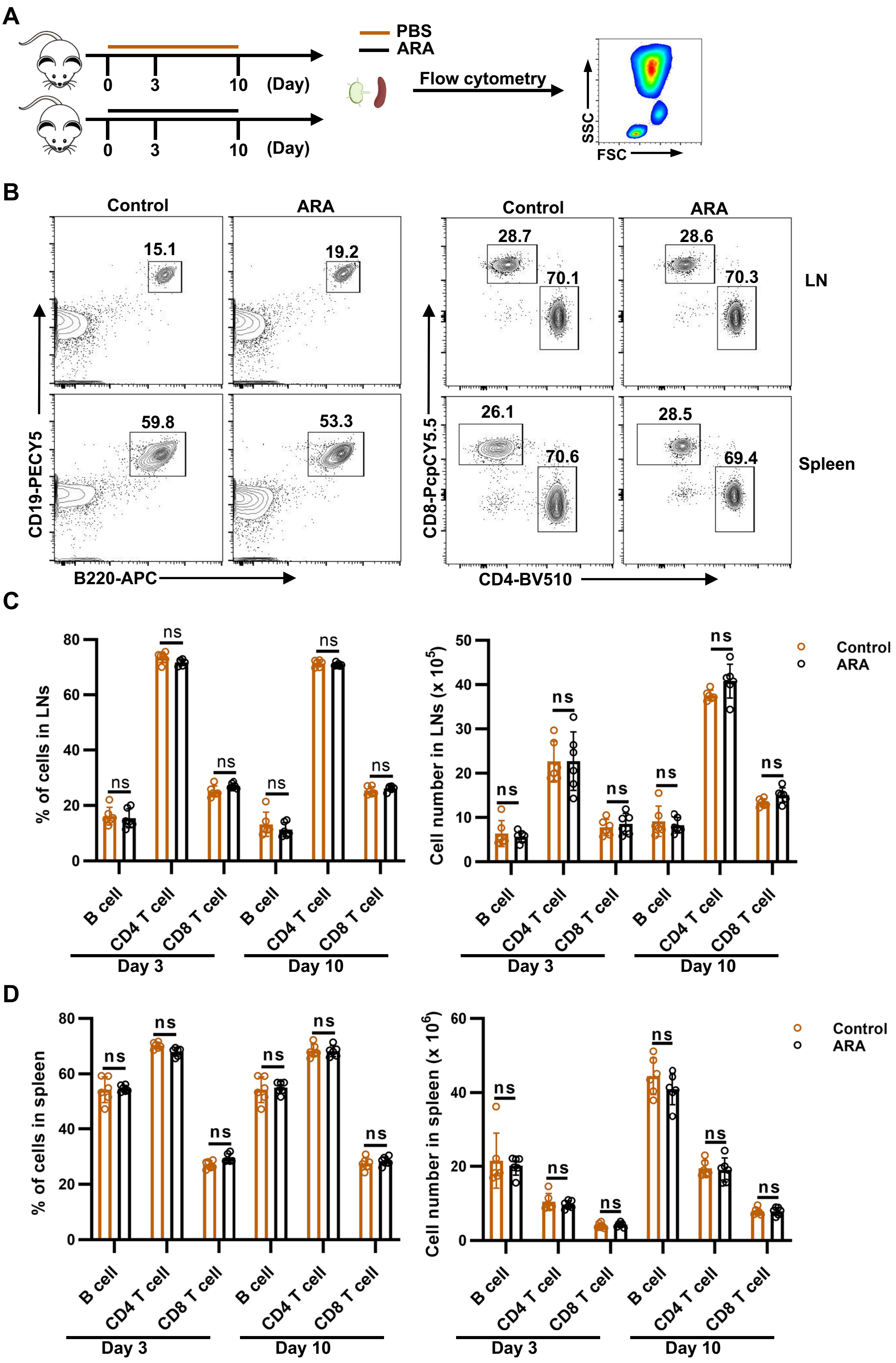
A
PBS
ARA
0 3 10 (Day)
0 3 10 (Day)
Flow cytometry
SSC
FSC
B
Control
ARA
15.1
19.2
59.8
53.3
CD19-PECY5
B220-APC
Control
ARA
28.7
70.1
28.6
70.3
LN
26.1
70.6
28.5
69.4
Spleen
CD8-PcpCY5.5
CD4-BV510
C
% of cells in LNs
Cell number in LNs (x 10^5)
Control
ARA
ns
B cell
CD4 T cell
CD8 T cell
Day 3
Day 10
D
% of cells in spleen
Cell number in spleen (x 10^6)

Figure EV2. The immune homeostasis was not influenced by daily ARA diet in mice spleen and lymph nodes.

(**A**) Schematic diagram of the study design. Mice supplemented with ARA for 10 days, and lymph nodes and spleen were collected on day 3 and day 10 for flow cytometry analysis, respectively. Mice supplemented with PBS as control. (**B**) Representative flow cytometry plots of lymph nodes (LNs) and spleen from mice supplemented with ARA for 3 days and 10 days to identify total B cells ($B220^{+}CD19^{+}$), CD4 T cells ($CD3^{+}$ $CD4^{+}$) and $CD8^{+}$ T cells ($CD3^{+}$ $CD8^{+}$). (**C**, **D**) Percentages and absolute cell counts of B cells, CD4 T cells and CD8 T in LNs (**C**) and spleen (**D**) from mice supplemented with ARA for 3 days and 10 days ($n = 6$). Data are representative of two independent experiments. Data are shown as mean ± SEM and each point represents an individual mouse. Significance was calculated by unpaired two-tailed *t* test; ns, no statistical significance.

© The Author(s)

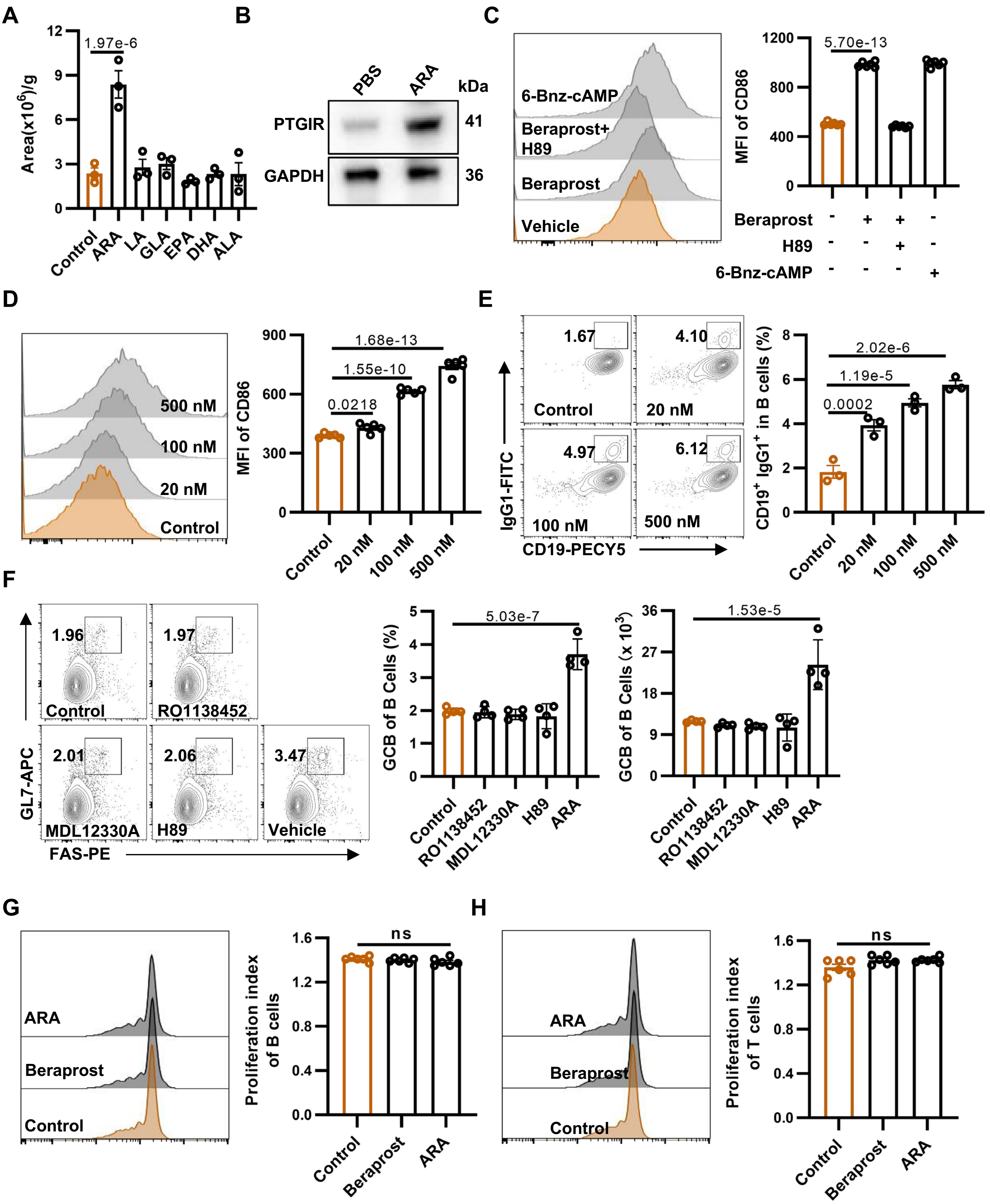
A
1.97e-6
Area(x10^6)/g
Control
ARA
LA
GLA
EPA
DHA
ALA
B
PBS
ARA
kDa
PTGIR
41
GAPDH
36
C
6-Bnz-cAMP
Beraprost+
H89
Beraprost
Vehicle
5.70e-13
MFI of CD86
Beraprost
H89
6-Bnz-cAMP
D
500 nM
100 nM
20 nM
Control
1.68e-13
1.55e-10
0.0218
MFI of CD86
E
1.67
4.10
4.97
6.12
Control
20 nM
100 nM
500 nM
IgG1-FITC
CD19-PECY5
2.02e-6
1.19e-5
0.0002
CD19+ IgG1+ in B cells (%)
F
1.96
1.97
2.01
2.06
3.47
Control
RO1138452
MDL12330A
H89
Vehicle
GL7-APC
FAS-PE
5.03e-7
GCB of B Cells (%)
1.53e-5
GCB of B Cells (x 10^3)
ARA
G
ARA
Beraprost
Control
ns
Proliferation index of B cells
H
ARA
Beraprost
Control
ns
Proliferation index of T cells

 © The Author(s)

Figure EV3. PGI_2 derived from ARA promotes CD86 expression and enhances the activity of AID in FO B cells.

(**A**) Quantification of PGI_2 from inguinal lymph nodes of mice supplemented with various PUFAs ($n = 3$). (**B**) Western Blotting analysis of PTGIR expression in lymph nodes of mice supplemented with ARA. (**C**) CD86 expression on activated B cells treated with vehicle, Beraprost, Beraprost plus H89, or 6-Bnz-cAMP alone ($n = 6$). Left: Representative flow cytometry plots. Right: Statistic data of the mean fluorescence intensity (MFI) of CD86. (**D**) Expression levels of CD86 on stimulated FO B cells following treatment with varying concentrations of Beraprost ($n = 5$). Left: Representative flow cytometry plots. Right: Statistic data MFI of CD86. (**E**) Flow cytometry analysis of the percentage of $CD19^+$ $IgG1^+$ B cells under stimulations of different concentrations of Beraprost ($n = 3$). Left: Representative flow cytometry plots. Right: Statistic data of the $CD19^+$ $IgG1^+$ B cells. (**F**) Flow cytometry analysis of GC B cells ($B220^+$ $GL7^+$ FAS^+) from mice supplemented with ARA under various inhibitors treatment on day 10 after immunization with OVA ($n = 4$). Left: Representative flow cytometry plots of GC B cells. Right: Statistic data of the percentages and cell numbers of GC B cells. (**G**, **H**) B Cell and T cell proliferation measured by CellTrace Violet (CTV) dye in LPS/IL-4-activated murine B cells treated with Beraprost (500 nM) and ARA (1 µM) ($n = 6$). Left: Representative flow cytometry plots. Right: Statistic data of the proliferation index of B cells and T cells. Data are representative of two or three independent experiments. All graphs represent mean ± SEM and all data points represent individual mice. Significance was calculated by one-way ANOVA with Tukey's multiple comparisons test; ns, no statistical significance.

© The Author(s)

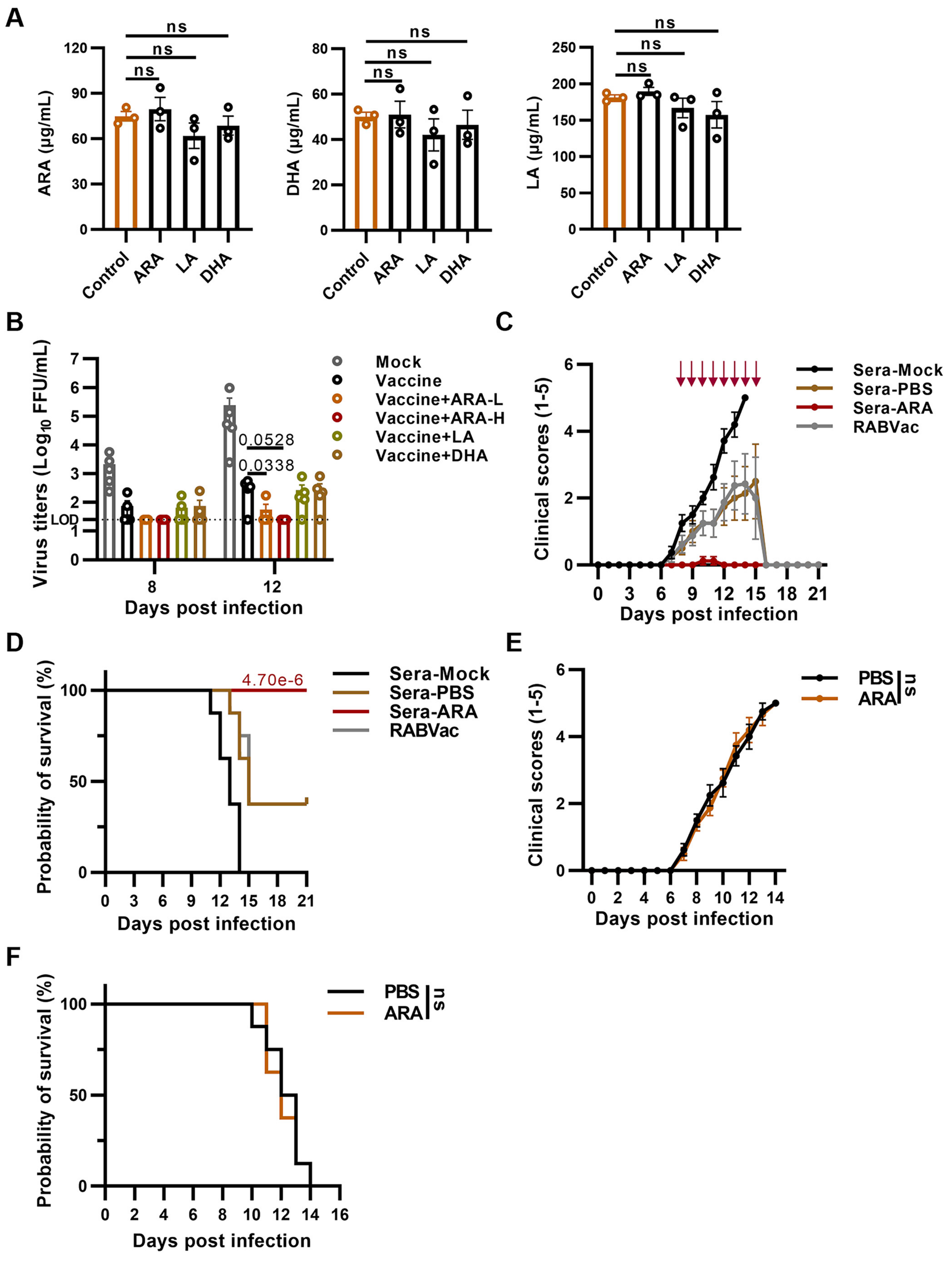
A
ns
ns
ns
ARA (μg/mL)
120
90
60
30
0
Control
ARA
LA
DHA
DHA (μg/mL)
80
60
40
20
LA (μg/mL)
250
200
150
100
50
B
Virus titers (Log10 FFU/mL)
LOD
0.0528
0.0338
Days post infection
Mock
Vaccine
Vaccine+ARA-L
Vaccine+ARA-H
Vaccine+LA
Vaccine+DHA
C
Clinical scores (1-5)
Sera-Mock
Sera-PBS
Sera-ARA
RABVac
D
Probability of survival (%)
4.70e-6
E
PBS
ARA
ns
F

 © The Author(s)

Figure EV4. Humoral immunity enhanced by ARA supplementation protects immunized mice against RABV challenge.

(**A**) Quantitative analysis of ARA, DHA and LA concentrations in murine plasma two weeks following cessation of dietary supplementation ($n = 3$). (**B**) Virus titers in mouse brain. The mice brains were collected at day 8 and day 12 post-infection and virus titers were calculated and expressed as focus-forming units per ml (FFU/mL) ($n = 5$). LOD, limit of detection. (**C**, **D**) Clinical scores and survival curves of mice that received immune sera before challenge ($n = 8$). The mice were intraperitoneally (i.p.) administrated with 200 µL sera. One day later, mice were i.m. challenged with 100 LD50 of RABV. Sera-Mock, the mice were received sera from mice without vaccination. Sera-PBS, the mice were received sera from immunized mice that supplemented with PBS. Sera-ARA, the mice were received sera from immunized mice that supplemented with ARA. RABVac, the mice were immunized with the inactivated rabies vaccine without receiving any sera. (**E**, **F**) Clinical scores and survival curves of mice received PBS or ARA ($n = 8$). The mice were administered with ARA (5 mg) or PBS control for 10 days. Fourteen days later, the animals were i.m. challenged with 100 LD_{50} of RABV, and clinical scores and survival were monitored. The arrows (**C**) indicate a significant difference between the group of Sera-PBS and Sera-ARA. Data are representative of two independent experiments. Data are shown as mean ± SEM and all data points represent individual mice. Significance was calculated by one-way ANOVA with Tukey's multiple comparisons test (**A**, **B**), unpaired two-tailed *t* test (**C**, **E**) and log rank (Mantel–Cox) test (**D**, **F**); ns, no statistical significance.

© The Author(s)

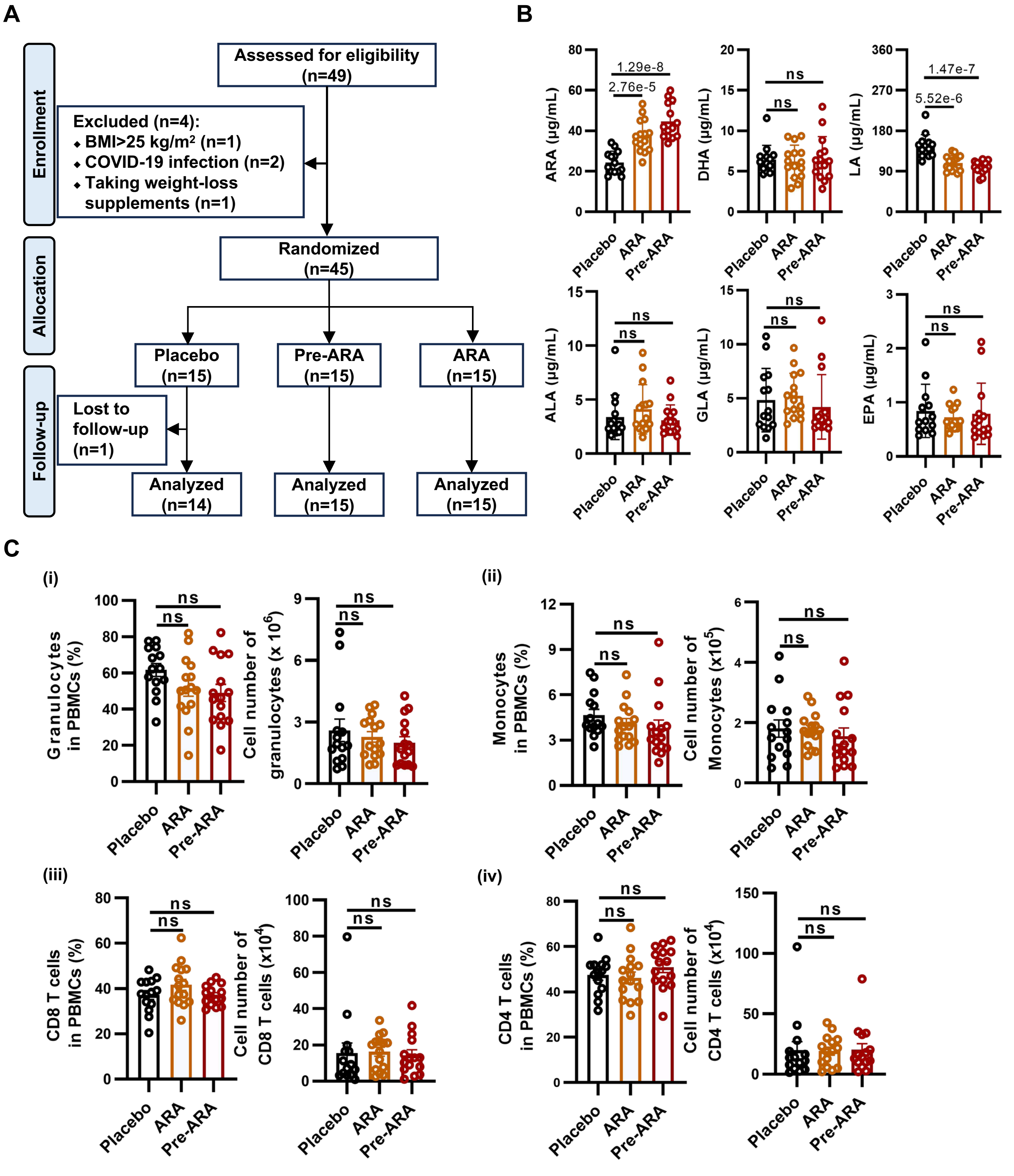
A
Enrollment
Allocation
Follow-up
Assessed for eligibility (n=49)
Excluded (n=4):
BMI>25 kg/m² (n=1)
COVID-19 infection (n=2)
Taking weight-loss supplements (n=1)
Randomized (n=45)
Placebo (n=15)
Pre-ARA (n=15)
ARA (n=15)
Lost to follow-up (n=1)
Analyzed (n=14)
Analyzed (n=15)
Analyzed (n=15)
B
ARA (μg/mL)
1.29e-8
2.76e-5
DHA (μg/mL)
ns
LA (μg/mL)
1.47e-7
5.52e-6
ALA (μg/mL)
GLA (μg/mL)
EPA (μg/mL)
Placebo
ARA
Pre-ARA
C
(i)
Granulocytes in PBMCs (%)
Cell number of granulocytes (x 10⁶)
(ii)
Monocytes in PBMCs (%)
Cell number of Monocytes (x10⁵)
(iii)
CD8 T cells in PBMCs (%)
Cell number of CD8 T cells (x10⁴)
(iv)
CD4 T cells in PBMCs (%)
Cell number of CD4 T cells (x10⁴)

 © The Author(s)

Figure EV5. The effects of ARA supplementation on PBMCs sourced from volunteers.

(**A**) Flow diagram of the study. Placebo, taking 512.4 mg sunflower seed oil daily ($n = 14$). Pre-ARA, taking 512.4 mg of ARA daily on day −3–13 ($n = 15$); ARA, taking 512.4 mg of ARA daily on day 0–13 ($n = 15$); (**B**) Quantitative analysis of PUFA concentrations in plasma obtained from volunteers supplemented with ARA ($n = 14$ in Placebo group; $n = 15$ in ARA and Pre-ARA group). (**C**) Statistic data of the percentages and cell numbers of granulocytes, monocytes, total lymphocytes, CD4 T cells, and CD8 T cells in the PBMCs on day 14 after the first shot immunization ($n = 14$ in Placebo group; $n = 15$ in ARA and Pre-ARA group). All graphs represent mean ± SEM, and all data points represent individual volunteers. Significance was calculated by one-way ANOVA with Tukey's multiple comparisons test; ns, no statistical significance.

© The Author(s)
